# Design method of blasthole charge structure based on lithology distribution

**DOI:** 10.1038/s41598-021-03758-y

**Published:** 2021-12-20

**Authors:** YingXian Chen, Jian Chen, PengFei Wang, Meng Zhou, HongXia Yang, JiaYing Li

**Affiliations:** College of Mining, Liaoning Technical University, Fuxin, 123000 China

**Keywords:** Fossil fuels, Civil engineering, Energy infrastructure

## Abstract

The density of geological exploration boreholes is one of the main bases for blasthole charge structure design. Due to the low density of geological exploration boreholes, it is impossible to obtain the blast hole rock formations' distribution accurately. With the development and application of intelligent drilling rigs, the lithology distribution data of the blasthole can be accurately obtained, and a blasthole charge structure design method based on the lithology distribution is proposed. The blasthole lithology data collected by the intelligent drilling rig is divided into 7 categories according to the rock hardness, and the adjacent strata with similar lithology are combined and divided into two groups of soft rocks and hard rocks. According to the rock stratum grouping data of the blasthole and the unit explosive consumption of each type of lithology, the explosive amount and charge length required for the soft rock group and the hard rock group can be calculated, respectively. Finally, the blasthole charge structure is designed according to the thickness and charge position of the hard rocks. With the C++ programming language, this method is realized and applied in the Shengli Open-pit Coal Mine of Inner Mongolia Autonomous Region of China. The application results show that, compared with the traditional hole charging structure design method, this method can realize accurate segmented charging of the hole, improve the blasting effect and the degree of rock fragmentation, and reduce the blasting cost.

## Introduction

When charge blasting is carried out in engineering, with the explosion of a whole section of explosives, the enormous initial pressure generated by the explosion product is much larger than the strength of the rock mass, and the intense shock wave begins to spread the medium and break it. Most of the explosive energy is consumed in the vicinity of the charge during continuous charge^1^. The rock mass cannot be further broken, and secondary blasting is needed. Therefore, the sectional charge is significant in improving the blasting effect and reducing the blasting cost.

As early as 1940, in order to change the effective transfer of the energy of the explosion mechanism to the solid medium, Mel'Nikov proposed for the first time that the energy of the explosion may be redistributed and the rock breaking in an inefficient working form to reduce the loss, and proposed the idea of segmented charge structure^2^. Later in 1954, Marchenko increased the energy utilization rate of explosives in blasting and proposed to fill the broken air gap of rock and the cavity of jet blasting, forming the prototype of the concept of segmented charges^3^.

**Figure 1:**
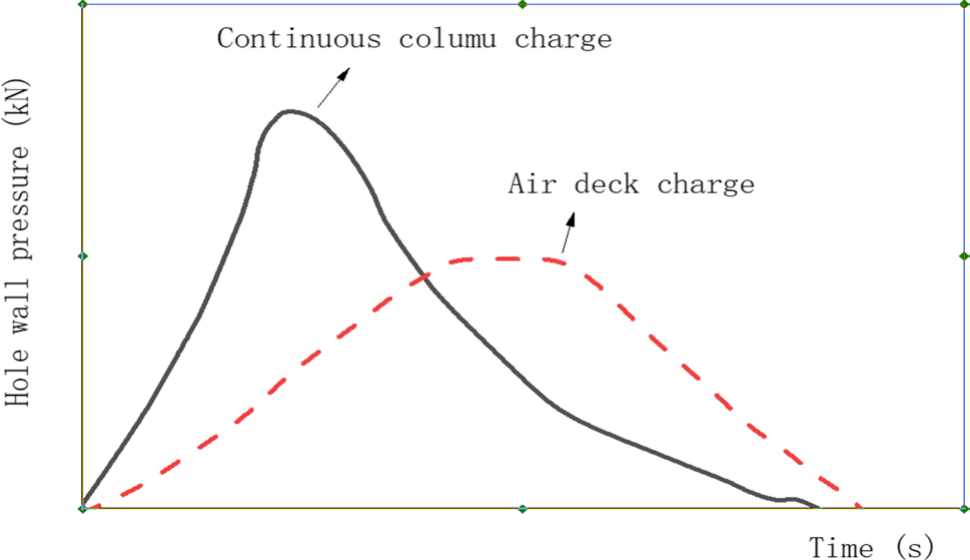
Hole wall pressure and action time^5^.

Afterward, some researchers carried out theoretical and model studies and studied its potential mechanism and impact on explosive performance, making the segmented charge further. In 1981, Fourney et al. proved that when the shock wave generated from the blasthole charge explosion reaches the blockage, it could be rebounded to strengthen the stress field, and the period of its action on the surrounding materials increased by 2–5 times, to achieve better blasting effects^4^. When the blasting filling material is air, the above influence is most apparent, the most widely used air interval charging technology in engineering. The change in hole wall pressure and time between continuous cylindrical and air charge is shown in Fig. 1.

In 1988, Bussey and Borg proposed a pre-separation technology of spacing charge at the 21st International Conference on Explosives and Blasting Technology^6^. In 1996, Liu L and Katsabanis reported on the influence of air-space charges on blasting at the Fifth International Conference on Blasting Stones^7^. Air-space charging technology has gradually entered the project, and it has achieved considerable development.

In 1993, some of the results presented by Moxon et al.'s assistive experiments on air-spaced charge blasting techniques showed^8^, the increase in charge coupling degree and explosion pressure does not necessarily lead to an increase in rock fragmentation^9^. So how to improve the degree of rock fragmentation in blasting engineering has become one of the research directions in recent years. In 2003, Lu and Hustrulid theoretically determined a reasonable air segmentation ratio to improve the effect of rock fragmentation^10^. In 2017, Eugie Kabwe proposed using airbags instead of air segments, which improved the impact efficiency and rock fragmentation^11^. In 2019, M. B. Hayata and L. Alaghab studied the optimum air charge position and volume, further improving the crushing effect and reducing the cost^12^.

With the development of intelligent technology in recent years, more and more intelligent air-spaced charge technologies have been proposed. For instance, in the segmented charge proposed by Bae Sang Hun and Bae Yong Cheol in 2013, multiple blasters were blasted at a time to reduce noise and vibration^13^; in 2018, Kim Jong in and Wi Jang Bok, et al. proposed sequential automatic explosive charge through non-electric detonators in segmented charge^14^, and in 2019, Choi Heon Kil and Moon Hong Pyo et al. proposed to guide the filling material of segmented charge to the inner wall of the blasting hole, so that the filling material had strong sealing force^15^.

How to combine intelligence with blasting and obtain an excellent blasting effect is the key to current development. However, due to technical limitations, these studies and applications mentioned above do not involveusing accurate lithology distribution of blastholes to carry out blasthole charge structure design. With the development and application of intelligent drilling rigs, it is possible to accurately obtain lithology distribution data in the blasthole by accurate lithology distribution to calculate the different lithology required to charge, which will provide a new method for the design of the blasthole charge structure.

## Principle and method of blasthole charge structure design

The use of segmented charge technology for blasting has become a very mature rock fragmentation technology. In this technology, the air gap is introduced into the explosive column by segmented charge, which is used as a common method to optimize rock fragmentation under a given charge length^16^.

**Figure 2:**
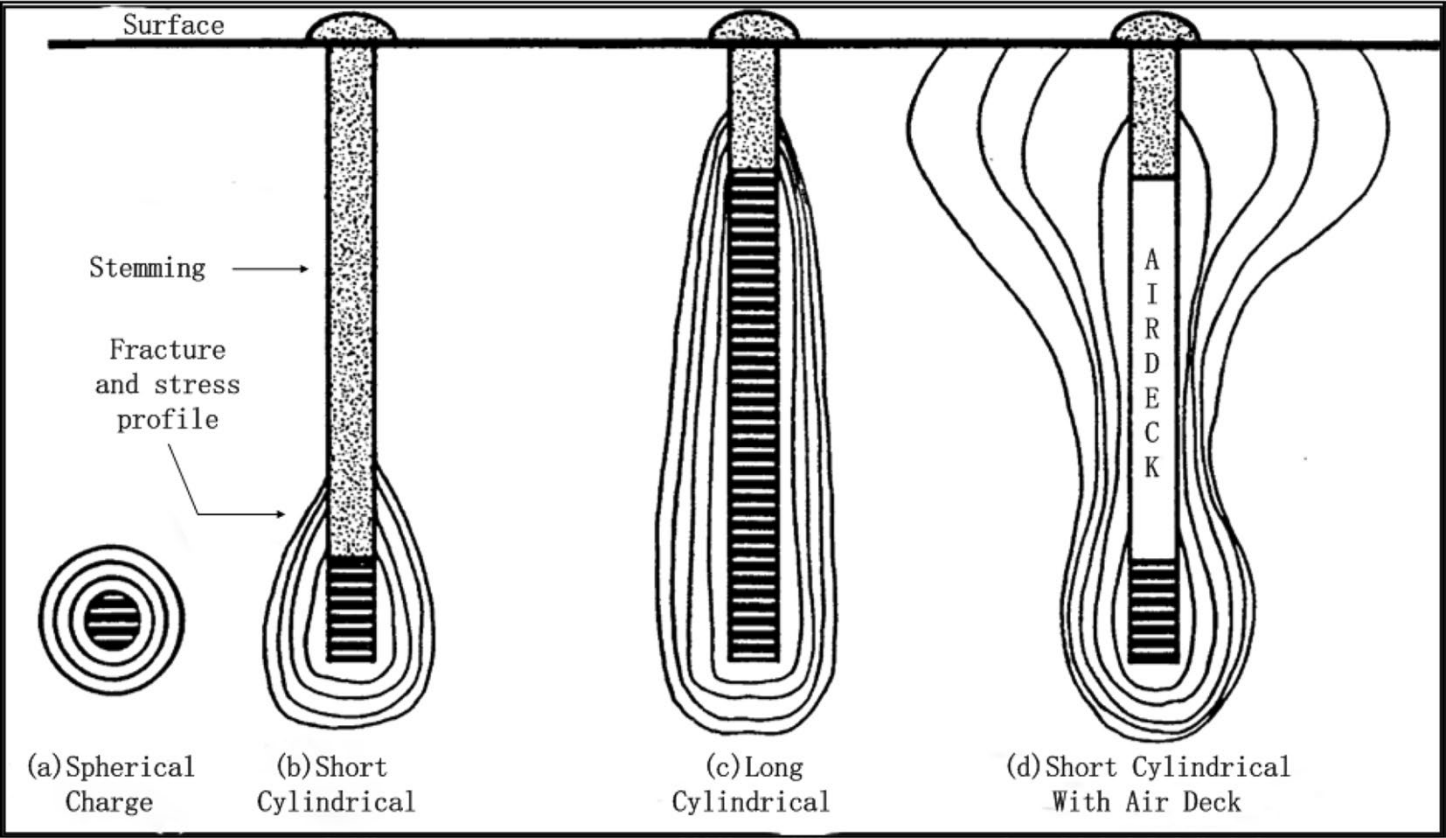
Fracture and stress profiles resulting from different charge geometries and distributions^18^.

In 1970, Mel'Nikov and Marchenko carried out field experiments using air explosives, and the results showed that the segmented charge is better than the traditional blasting method^17^. Nevertheless, there is no discussion about the shock waves generated from the explosion. It was not until 1987 when the air gap's air retention and airtightness problems in air-space charge were discussed at the 2nd International Blasting and Rock Fragmentation Symposium by Chiappetta and Memmele^18^, the problem of the air gap entered everyone's mind. The air gap made the gas generated by the explosion move and expand into the air gap, thereby reducing the initial drilling pressure. Shock waves oscillate and influence each other in the blasthole, and interact with the riser string and the bottom of the blasthole. Repeated interaction results in enhanced secondary shock fronts and allows the shock waves to act on surrounding rock mass for a more extended period. The fracture and stress profiles resulting from different charge geometries and distributions are shown in Fig. 2.

We mainly study the structure of blasthole air-space charge. Compared with complete charge, the advantages of segmented charge are mainly reflected in the following points:

1. To separate one blast into multiple blasts to improve the crushing effect;
2. To reduce the explosive charge;
3. To form more shock wave interactions or even shock collisions;
4. To separate one blast into multiple blasts^19^.

Deck charges can be divided into two types:

1. Different explosives are charged in a single hole, and each explosive is charged in one part of the blasthole. These different decks of explosives can be charged one over the other without air gaps between them.
2. One explosive is charged in a single hole. However, it is separated into several parts using stemming. Stemming can be any material.

In this article, we mainly discuss the second segmented charge type.

**Figure 3:**
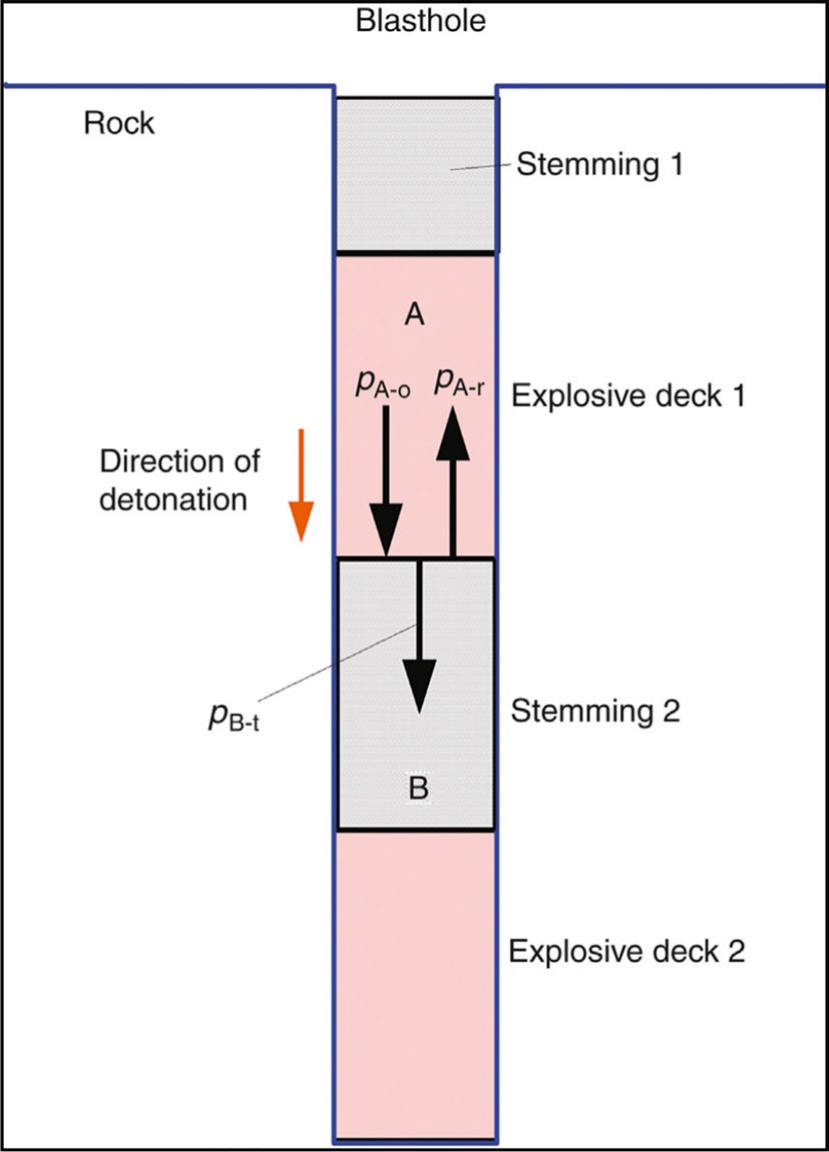
Shock wave propagation from explosive to stemming^20^.

The air gap charge is to make full use of the air between the blastholes to interact with the shock waves generated during the explosion. As shown in Fig. 3, when a shock wave travels from material $A$ with lower impedance $Z_{A}$ to material $B$ with higher impedance $Z_{B}$, the pressure $P_{{B{\text{ - t}}}}$ of the shock wave caused by material $B$ (or called transmitted wave) will be greater than the pressure $P_{A\text{-o}}$ of the original shock wave in $A$; that is,

$$ P_{B\text{-t}} > P_{A\text{-o}} $$

Shock wave $P_{A\text{-o}}$ originating from detonation is often compressive, so is the transmitted shock wave $P_{B\text{-t}}$. Because on the interface between $A$ and $B$ the shock pressure should be same, the reflected shock wave $P_{A\text{-r}}$ is also compressive. As a consequence, the compressive $P_{A\text{-r}}$ and the remained compressive $P_{A\text{-o}}$ will effectively superimpose together and form a stronger shock wave. This stronger shock wave will travel back to A and may result in one more time of wave interaction at the interface between $A$ and stemming 1. Finally, the shock wave action in the blasthole is strengthened and also prolonged. This should, of course be useful for rock fracture and fragmentation^20^.

According to the charge structure design principle and the lithology distribution of the blasthole obtained via the intelligent drilling rig, we could change the charge structure based on the distribution of soft rock and hard rock in the blasthole to calculate the explosive charge distribution of the charge section. Compared with the traditional charge structure, the blasting effect is improved, and the blasting cost is reduced.

## Method based on precise lithology distribution

In the process of rock blasting, the physical and mechanical properties of the rock are undoubtedly one of the main factors that determine the effect of rock blasting^21^. There is often a vague understanding that the harder the rock, the harder it is to be blasted in the current engineering world. The calculated unit consumption of explosives increases with the increase of rock hardness. However, the blasting effect is not exactly as expected in engineering practices. Instead, it is often found that the hardness of rock is not large while the unit consumption of explosives is large^22^.

According to the breaking mechanism of rock blasting^23^, the energy released by explosives acts on rocks by the shock wave and expansion pressure of detonation gas when an explosive explodes on rocks. The ultimate destruction of rocks is caused by the action stress produced by the explosion exceeding its strength limit, which indicates that the crushing state is closely related to the explosion energy and the mechanical properties of the rock. In other words, under the specific conditions of unit explosive consumption form, type, and free surface, the charge quantity is mainly determined by the mechanical properties of rock to achieve a specific crushing effect. Thus, the lithology distribution is considered the primary basis for determining the unit consumption of explosives^24^.

In an in-depth study of the lithology of hard rock, it is found that high stress has a significant impact on the blasting process and rock breaking effect. The results also show that high-site stress unloading and blasting loading are not two independent processes, on the contrary, they work in synergy, and the dynamic unloading has a significant effect^25^. Therefore, the distribution of hard rock is the key to the design of a segmented charge for blasthole.

### Grouping of blasthole rock strata

In the traditional blasting process, the lithology difference along the blast hole depth is not considered in the blasting structure. Therefore, a single charge structure was adopted. In the layered rock mass with soft and hard inclusions, the weak interlayer's compression and tensile resistance strength are extremely low compared with that in the complex rock formations, almost negligible. At that time, the weak interlayer can quickly become the overflow channel of the detonation gas and weaken the crushing and throwing effect of the high-pressure gas generated by the explosion. On the other hand, in the case of large hard rock without explosion cracks, it is challenging to break sizeable rocks by using the secondary crushing principle of block rock collision in the throwing process due to a weak interlayer^26^. Therefore, the division of soft rock and hard rock is an essential step of this design method.

**Figure 4:**
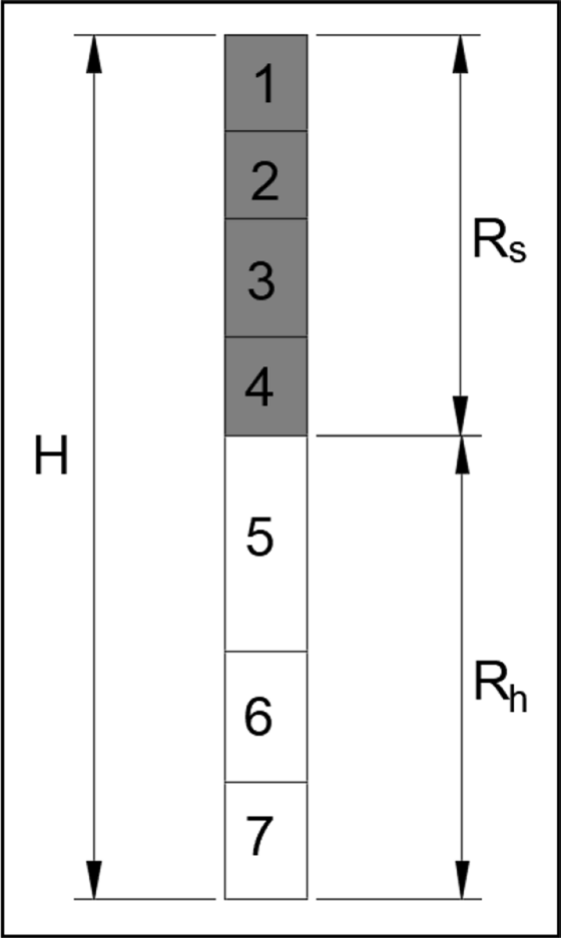
Length of blasthole charge.

The lithology data of the corresponding blastholes are obtained by intelligent drilling rigs. According to the hardness of the lithology, the blasthole rock strata are divided into 7 categories, and lithology classification numbers range from Type 1 to Type 7^27^. In order to facilitate the design and calculation of blasthole charge structure, the adjacent strata with similar lithology are combined and grouped. The lithology is divided into two groups of soft rock and hard rock, denoted as $s$ and $h$, respectively. The classification rule of soft rock and hard rock is that the strata numbered 1, 2, 3, and 4 are soft rocks, and rock strata numbered 5, 6, and 7 are hard rocks. The grouping diagram of blasthole rock strata is shown in Fig. 4: *H* refers to the blasthole depth, *R*_*s*_ means the thickness of soft rock, and *R*_*h*_ means the thickness of hard rock.

Based on the rock stratum grouping data of the blasthole and the unit explosive consumption of each lithology, the explosive quantity Q_*s*_ required by the soft rock group *s* and the explosive quantity Q_*h*_ required by the hard rock group *h* are calculated respectively. The sum *Q* of the explosive quantity required by each group is the charge quantity of the blasthole. The calculation formula is as follows^28–30^:

1 $$ Q_{s} = a\sum\limits_{i = 1}^{k} {q_{i} l_{i} } $$

2 $$ Q_{h} = a\sum\limits_{i = 1}^{k} {q_{i} l_{i} } $$

3 $$ Q = Q_{S} + Q_{{\text{h}}} $$

In the above formula, *a* is the affected area of the single blasthole; *q*_*i*_ is the unit explosive consumption of the i-th layer; *l*_*i*_ is the thickness of the i-th layer.

**Figure 5:**
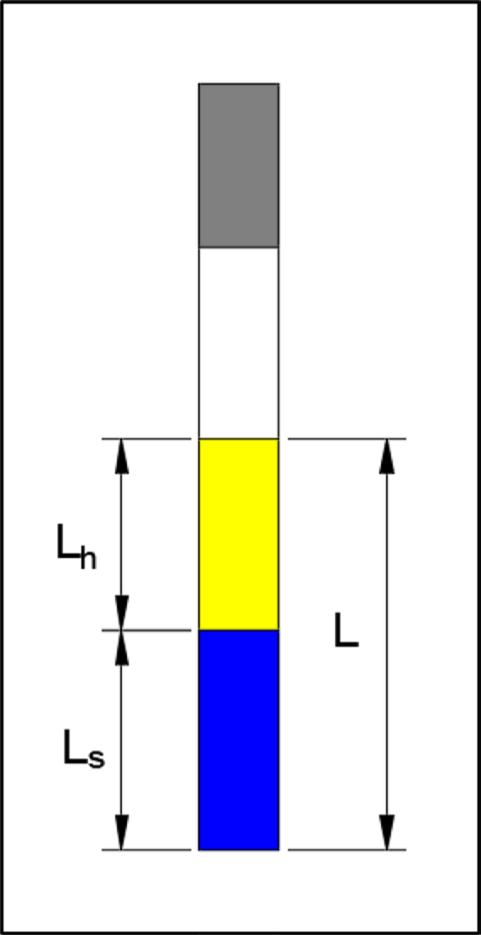
Classification of blasthole lithology.

Then, the charge lengths required for the soft rock group and hard rock group are calculated, respectively. The charge length of the soft rock group is *L*_*s*_, the charge length of the hard rock group is *L*_*h*_, and the total charge length *L* of the blasthole is the sum of the charge lengths of the soft rock group and hard rock group. The schematic diagram of blasthole charge length is shown in Fig. 5.

The calculation formula is as follows^31^:

4 $$ L_{s} = 4Q_{s} /(r\pi d^{2} ). $$

5 $$ L_{h} = 4Q_{h} /(r\pi d^{2} ) $$

6 $$ L = L_{{\text{s}}} + L_{h} $$

In the formula; *d* is the diameter of the blasthole; *r* is the volumetric weight of the explosive.

### Structural design of blasthole charge

To increase the operability of the on-site charge, the blasthole charge is divided into two-stage charge structure arrangement at most, so there are two kinds of charge structure of the blasthole. One is a single-stage charge, and the other is a two-stage charge. The division of charge structure is determined by the thickness and charge position of the hard rock. Suppose *H* is the total depth of the blasthole, *H*_*tmin*_ is the minimum filling length of the orifice, *H*_*s*_ is the charge depth of the soft rock, and *H*_*h*_ is the charge depth of the hard rock.

Thereinto, the calculation formula of the minimum filling length *H*_*tmin*_ of the blasthole orifice is as follows^32^:

7 $$ H_{t\min } = (15\sim 20)d $$

In the formula, *d* is the diameter of the blasthole. When the rock is hard, the plugging material contains much water, and the resistance line is larger, the larger value is adopted.

**Figure 6:**
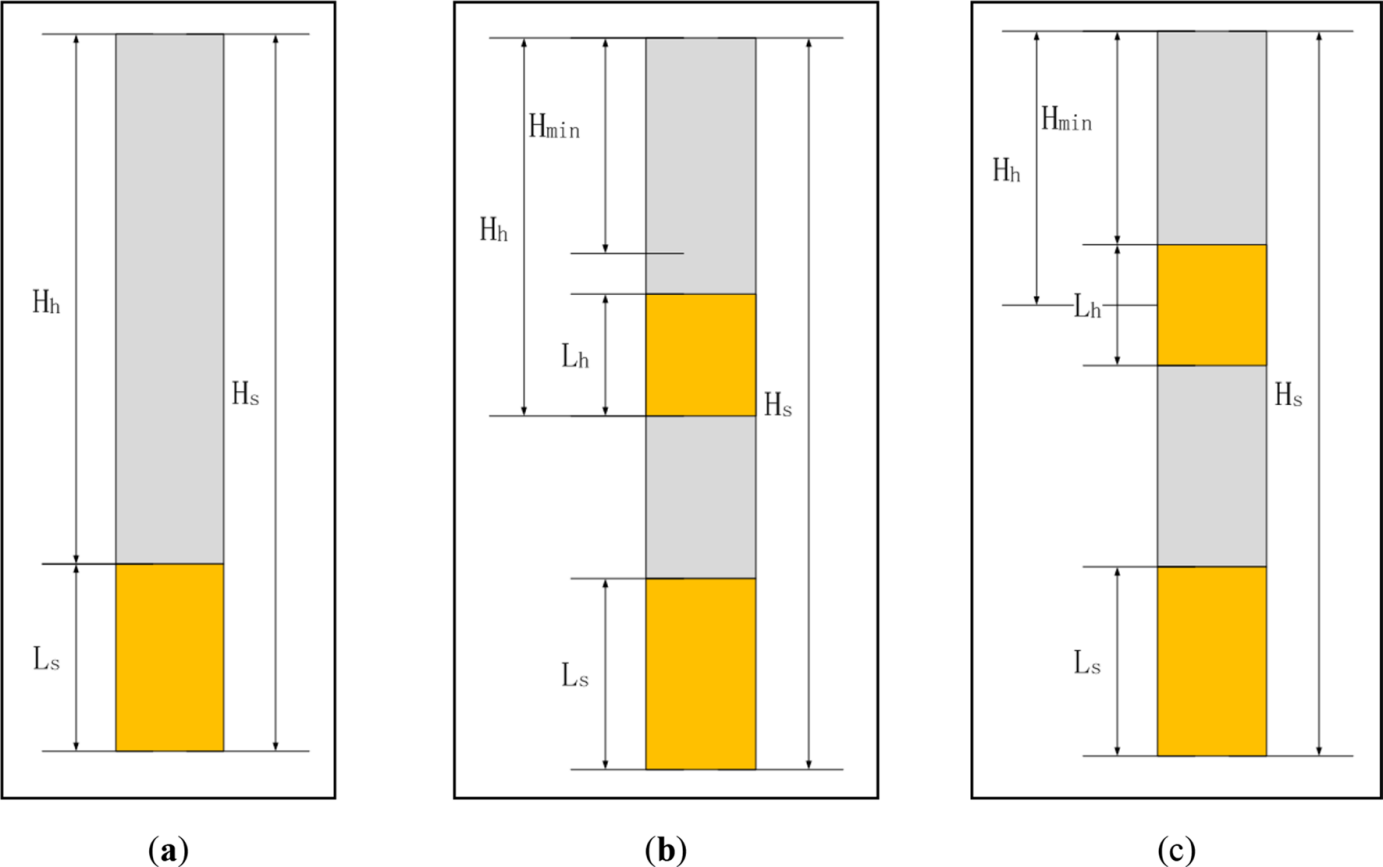
Charge structures in three cases.

The explosives in the soft rock section are charged from the bottom of the blasthole, so the charge depth of the soft rock *H*_*s* =_ *H*, and the initial hard rock charge depth *H*_*h*_ is determined by the location of the hard rock. The charge structure method is divided into three cases, as shown in Fig. 6:

1. If $H_{h} \ge H_{s} - L_{s}$ , it should be the single-stage charge, the charge depth is *H* and the charge length is *L*, as shown in Fig. 6a;
2. If $H_{h} < H_{s} - L_{s}$, and $H_{h} - L_{h} \ge H_{t\min }$, the charges are divided into two stages of the soft rock and hard rock charges respectively, as shown in Fig. 6b;
3. If $H_{h} - L_{h} < H_{t\min }$ , it takes a two-stage charge: the charge of soft rock is from the bottom of the blasthole, the hard rock charge depth is $H_{h} = H_{t\min } + L_{h}$, and the charge length is *L*_*h*_, as shown in Fig. 6c.

### Calculation of blasthole segment charge

Calculate the charge volume and charge length of the blasthole charge and determine the charge position and size parameters of the blasthole segment charge, including the hard rock charge depth *H*_*h*_ and the filling length *H*_*mid*_ of intermediate air. The calculation formulas are as follows:

8 $$ H_{h} = \left\{ {\begin{array}{*{20}c} {H_{h} ,{\text{when}}H_{h} < H_{s} - L_{s} ,{\text{and}}H_{h} - L_{h} \ge H_{t\min } } \\ {H_{t\min } + L_{h} ,{\text{when}}H_{h} - L_{h} < H_{t\min } } \\ \end{array} } \right. $$

9 $$ H_{mid} = H_{s} - L_{s} - H_{h} $$

Finally, the calculation results shall be generated into a blasthole charge table, and the blasthole can be charged in sections according to this table.

## Field tests

**Figure 7:**
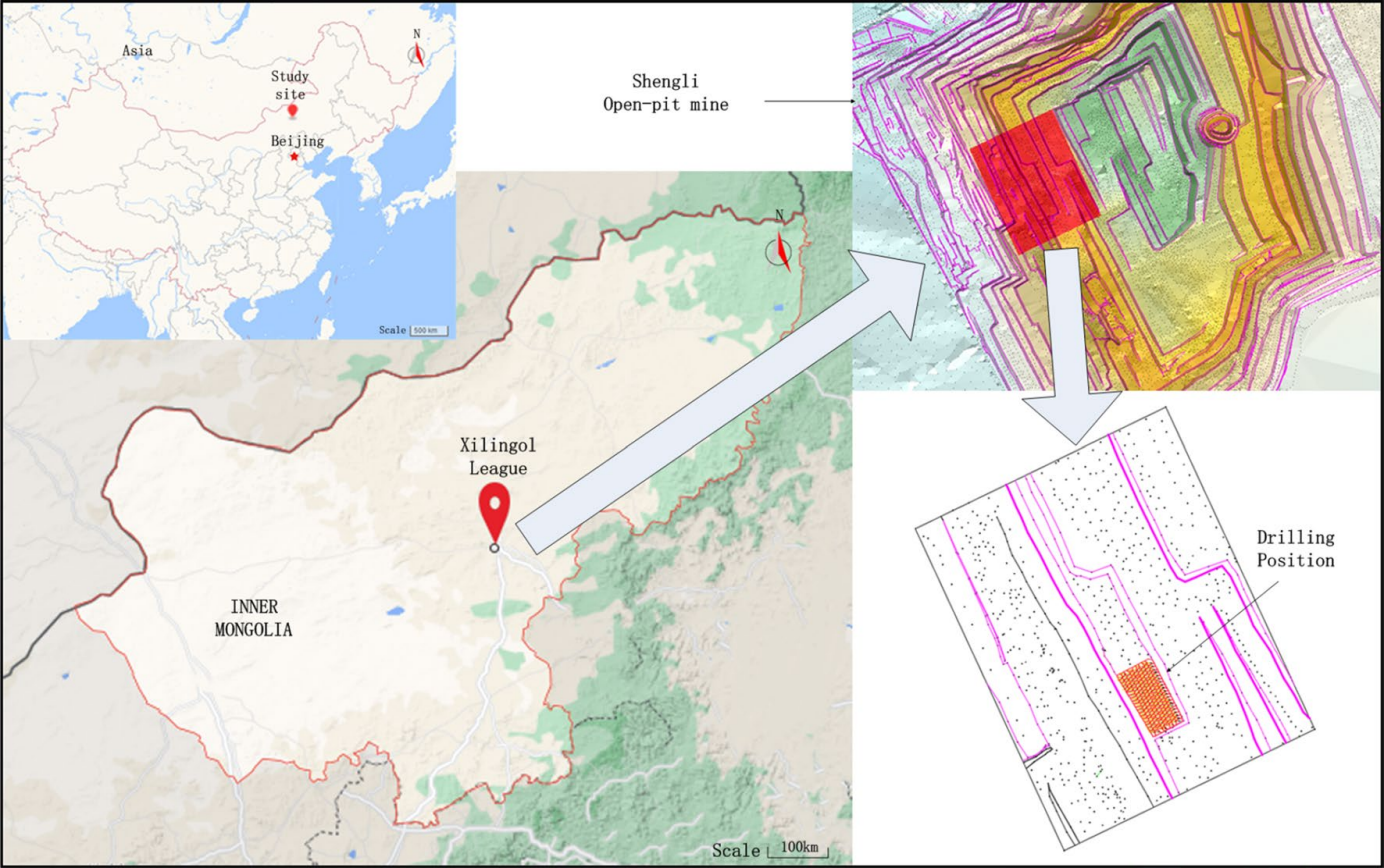
Location of the open-pit coal mine and the blasting area (The map used was downloaded from Google Maps (https://www.google.com/maps), where the pictures are combined with Visio(Microsoft Office Visio 2013) (https://www.microsoft.com/zh-cn/microsoft-365/previous-versions/microsoft-visio-2013)).

**Figure 8:**
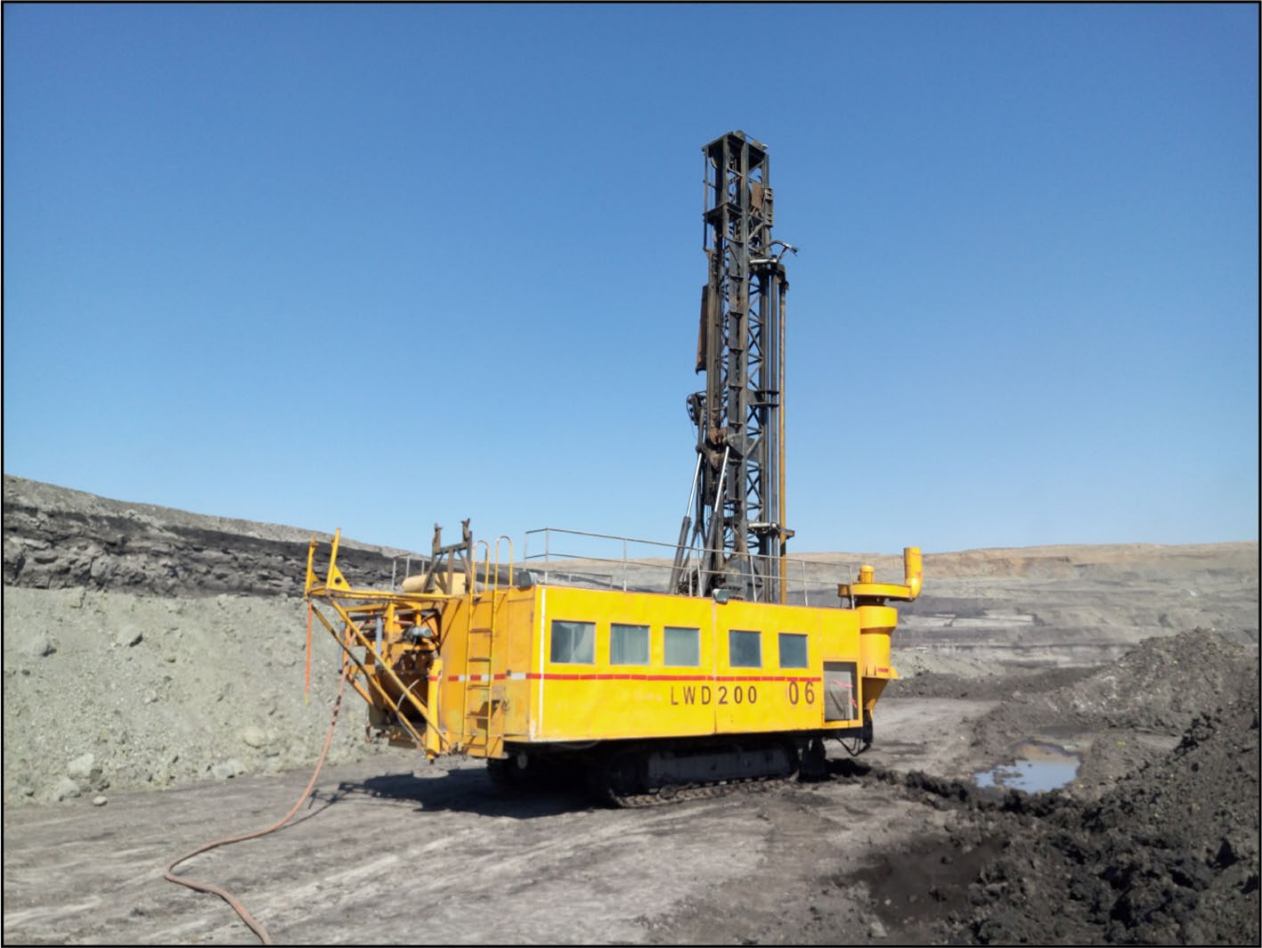
Working site of LWD-200B hydraulic drilling rigs.

**Figure 9:**
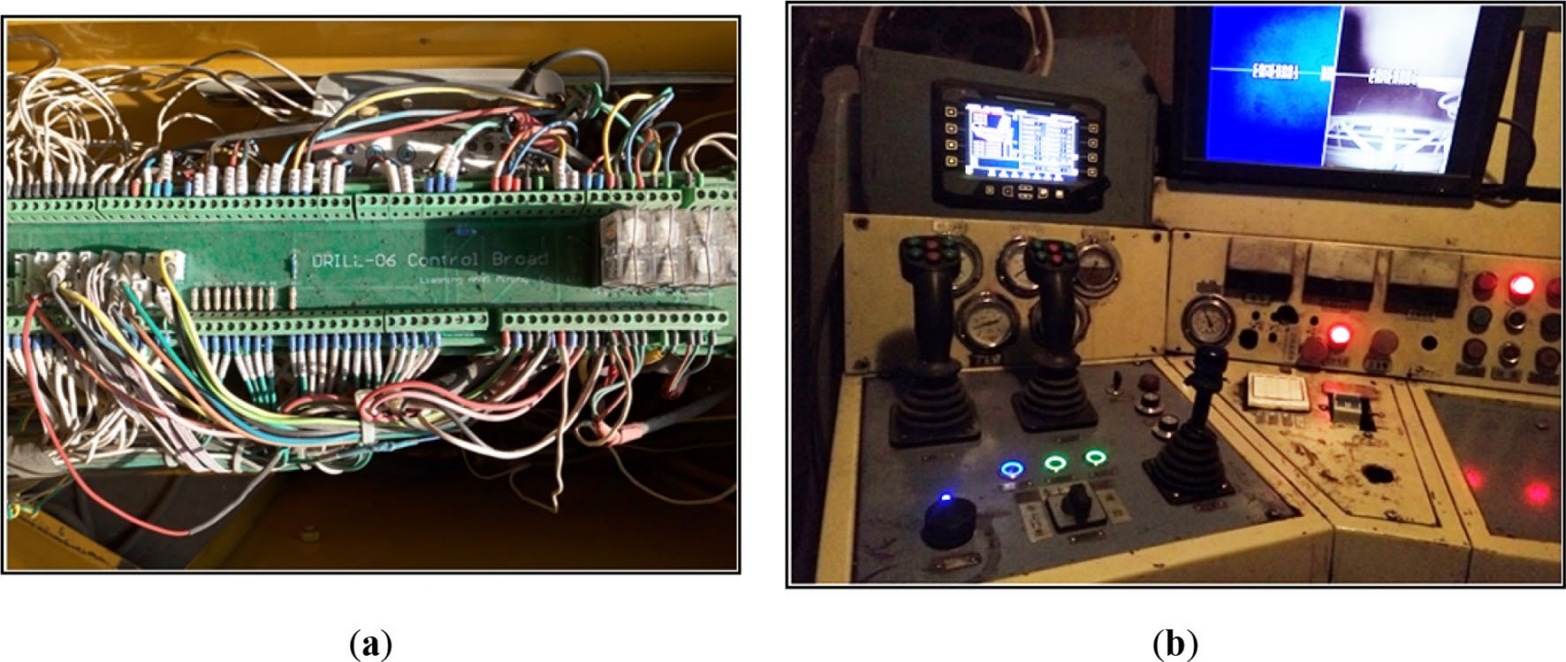
Intelligent lithology recognition system.

The application is located in the Xilinhot Shengli Open-pit Coal Mine of Inner Mongolia Autonomous Region, northeast China. The location of the open-pit coal mine and the blasting area is shown in Fig. 7. Using the LWD-200B hydraulic drilling rig to identify blasthole lithology, 165 groups of blastholes were obtained. The working site of the LWD-200B hydraulic drilling rig is shown in Fig. 8. The intelligent identification system of the drilling rig is shown in Fig. 9.

The first 6 rows of the data collected from the intelligent drilling rig recorded the blasthole number, rig status, rig number, start-up time, longitude, latitude, and elevation, respectively. Lines 7–15 were the records of a section of rock pillars. These data include blasthole number, blasthole depth, rotation speed, rotation pressure difference, pressurization pressure 1, pressurization pressure 2, drilling speed, wind suppression, and lithology identified. Subsequently, this record data will be circulated for each rock pillar until the blasthole is completed. The data listed in Table 1 lists the data of a section of rock pillar with blasthole number 0620171118170059 collected by No. 9 intelligent drilling. These data constitute the blasthole data files. Nevertheless, these data files are not convenient for management and further application. Therefore, it is necessary to establish a database to store and manage this data.

**Table 1:** Blasthole data.

"9";"1. blasthole number: 0620171118170059 "
"9";"2. rig number: 9"
"9";"3. start-up time: 20,171,118,170,059 "
"9";"4. longitude: 115°59′52.0 ″"
"9";"5. latitude: 44°0′2.0 ″"
"9";"6. elevation: 940.46 m"
"9";"7. blasthole number:0620171118170059 "
"9";"8. blasthole depth: 401 cm"
"9";"9. rspeed: 120 r/min"
"9";"10. rpress: 62 bar"
"9";"11. force 1: 43 bar"
"9";"12. force 2: 7 bar"
"9";"13. drillv: 4 cm/s"
"9";"14. wind suppress: 4 bar"
"9";"15. lithology identified: 1"

**Figure 10:**
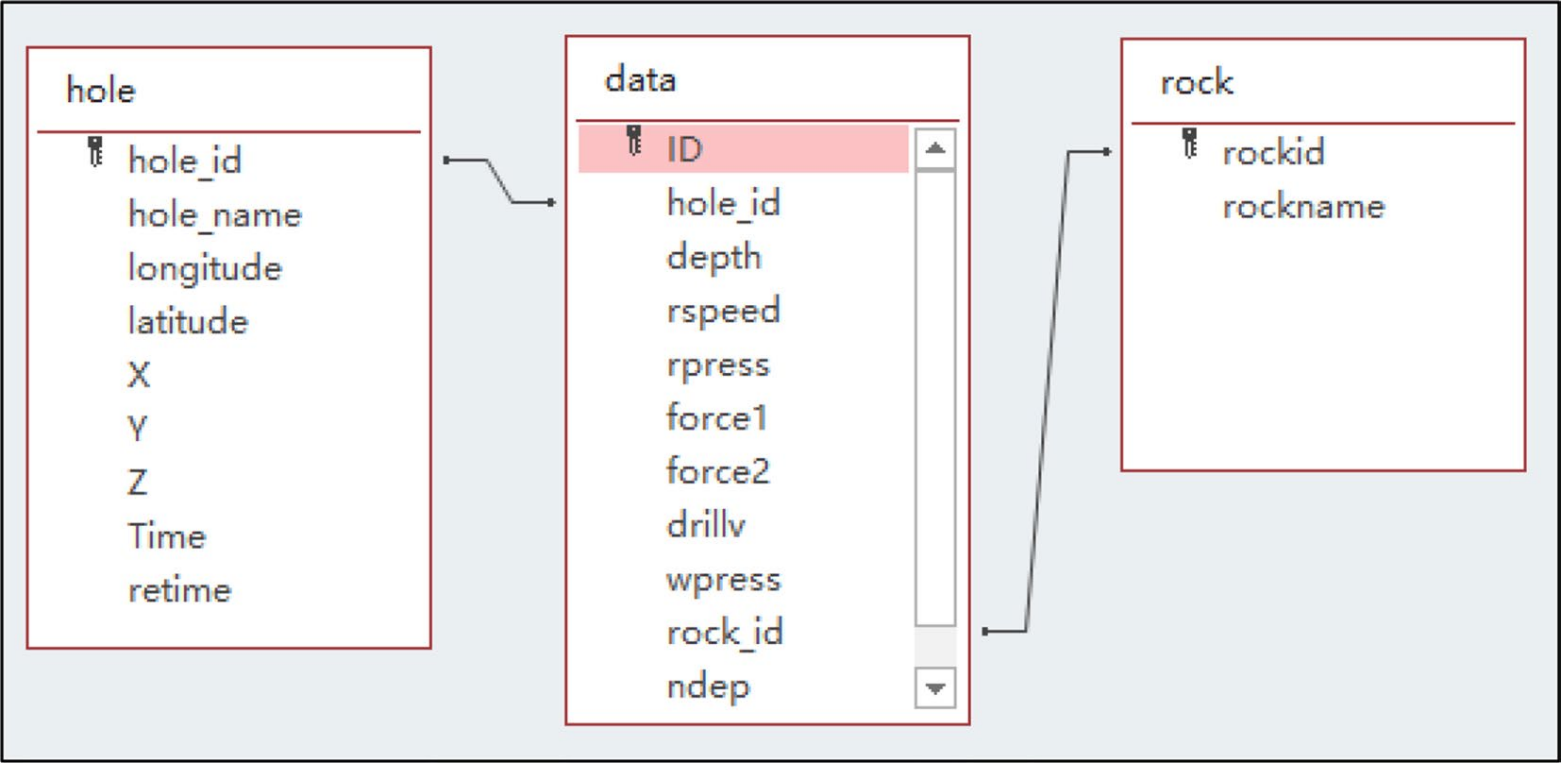
The relationship between the data tables.

**Figure 11:**
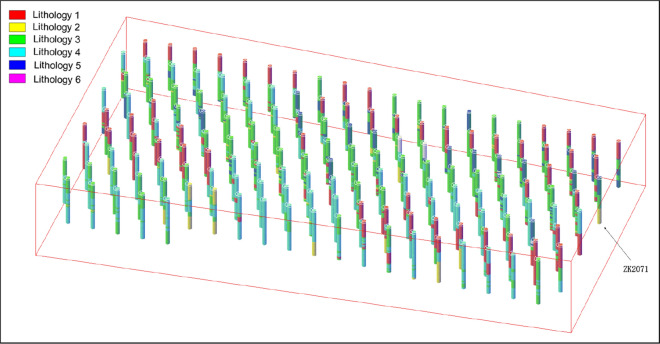
Three-dimensional columnar structure of all blastholes.

Use Microsoft Access to establish a blasthole database, and create three data tables in the database: blasthole table (*hole)*, blasthole data table *(data),* and lithology table *(rock)*, which are used to store data collected from the intelligent drilling rig. The relationship between the three data tables is shown in Fig. 10. Table *hole* is associated with Table *data* through hole_id, and Table *rock* is associated with Table *hole* through rock_id. The obtained lithology distribution data table of the blasthole is shown in Table 2. According to the lithology distribution data, we draw a three-dimensional histogram of all blastholes, as shown in Fig. 11. The different colors of the blasthole pillars in the figure indicate different rock strata.

**Table 2:** Lithology distribution data of blastholes.

hole_id	depth (m)	rspeed (r/s)	rpress (MPa)	force1 (MPa)	force 2(MPa)	drillv (m/s)	wpress (MPa)	rock_id	ndep	time
ZK2023	4.68	75	46	30	7	3	4	3	1	2017/11/6 11:15:28
ZK2023	5.04	105	55	40	8	7	4	3	2	2017/11/6 11:15:28
ZK2023	5.37	120	39	30	7	3	4	3	3	2017/11/6 11:15:28
ZK2023	5.74	90	57	48	8	6	5	3	6	2017/11/6 11:15:28
…	…	…	…	…	…	…	…	…	…	…
ZK2187	7.97	105	81	50	6	7	4	3	9	2017/11/22 14:21:53
ZK2187	17.49	105	89	63	5	7	5	4	10	2017/11/22 14:21:53

We are taking the lithology distribution data of the ZK2071 of the open-pit coal mine as an example to achieve the precise charge structure design of the blasthole.

### Blasthole rock formation grouping

**Figure 12:**
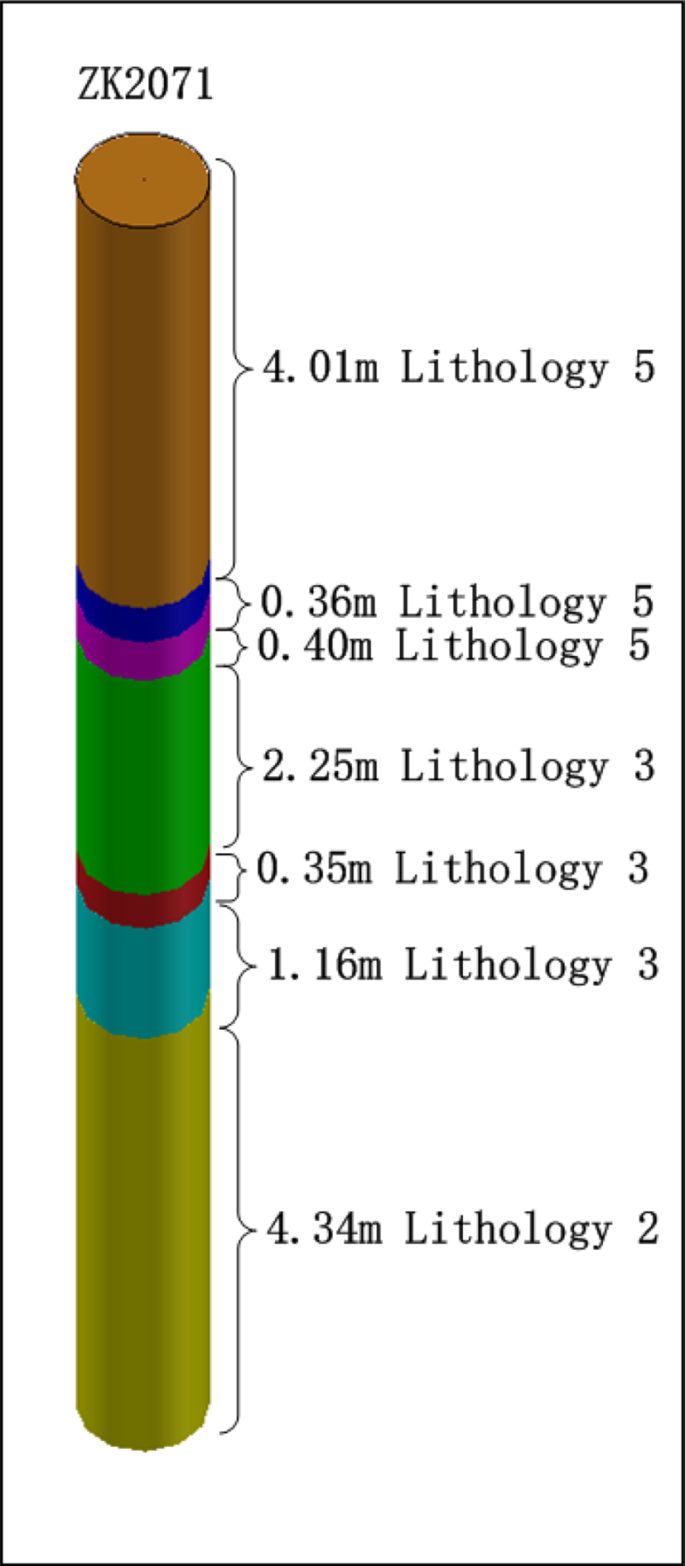
Rock classification map of blasthole ZK2071.

Based on the lithology distribution data of the blasthole ZK2071, the blasthole lithology is grouped. The lithology distribution data of the blasthole ZK2071 are shown in Table 3. There are 7 rock layers in blasthole ZK2071, and the lithology classification numbers are 5, 5, 5, 3, 3, 3, and 2. The three strata with lithology number 5 are divided into the hard rock group *h*, and the other four strata with lithology numbers 3 and 2 are divided into the soft rock group *s*. The blasthole depth *H* is 12.87 m, the soft rock thickness *R*_*s*_ is 8.1 m, and the hard rock thickness *R*_*h*_ is 4.77 m. The lithology distribution of blasthole ZK2071 is shown in Fig. 12.

**Table 3:** Lithology distribution data.

hole_id	Rock thickness(m)	Lithology	rock group	Unit explosive consumption (kg/m^3^)	Affected area of single blasthole (m^2^)
ZK2071	4.01	5	h	0.19	47.24
ZK2071	0.36	5	h	0.19	47.24
ZK2071	0.40	5	h	0.19	47.24
ZK2071	2.25	3	s	0.17	47.24
ZK2071	0.35	3	s	0.17	47.24
ZK2071	1.16	3	s	0.17	47.24
ZK2071	4.34	2	s	0.16	47.24

### Calculation of blasthole charge amount and length

1. Calculate the blasthole charge amount.
  According to the rock stratum grouping data of the blasthole and the unit explosive consumption of each lithology, the explosive quantity *Q*_*s*_ required for the soft rock group and the explosive quantity *Q*_*h*_ required for the hard rock group are calculated. The sum *Q* of the explosive quantity required for each group is the charge quantity of the blasthole. Put the data in the table into Formula (1) and (2) respectively and calculate that the explosive quantity *Q*_*s*_ required for the soft rock group is 63 kg, the explosive quantity *Q*_*h*_ required for the hard rock group is 42.81 kg, and the sum *Q* of explosive quantity required for each group is 105.81 kg according to Formula (3).
2. Calculate the length of the blasthole charge.
  The blasthole diameter *d* of the blasthole ZK2071 is 0.2 m, and the volumetric weight of explosive *r* is 1.60 × 10^3^ kg/m^3^. The charge length *L*_*s*_ of the soft rock group is 1.25 m calculated via blasthole charge length Formula (4). In the same way, according to Formula (5), the charge length *L*_*h*_ of the hard rock group is 0.85 m.
3. Structure design of blasthole charge.
  In this example, the total depth of the blasthole *H* is calculated to be 12.87 m, the minimum filling length range *H*_*tmin*_ is 3 m according to Formula (7), the depth of soft rock charge *H*_*s*_ is 12.87 m. Since the location depth of hard rock is 4.77 m, the depth of hard rock charge *H*_*h*_ is preliminary determined to be 4.77 m. Moreover, because the charge length of the soft rock group *L*_*s*_ is 1.25 m, and the charge length of the hard rock group *L*_*h*_ is 0.85 m, it is in line with the second charge situation mentioned above according to the judgment method that $H_{h} < H_{s} - L_{s}$, the filling length of the orifice should be 3.92 m (Stemming 1).

### Calculation of blasthole segment charge

**Figure 13:**
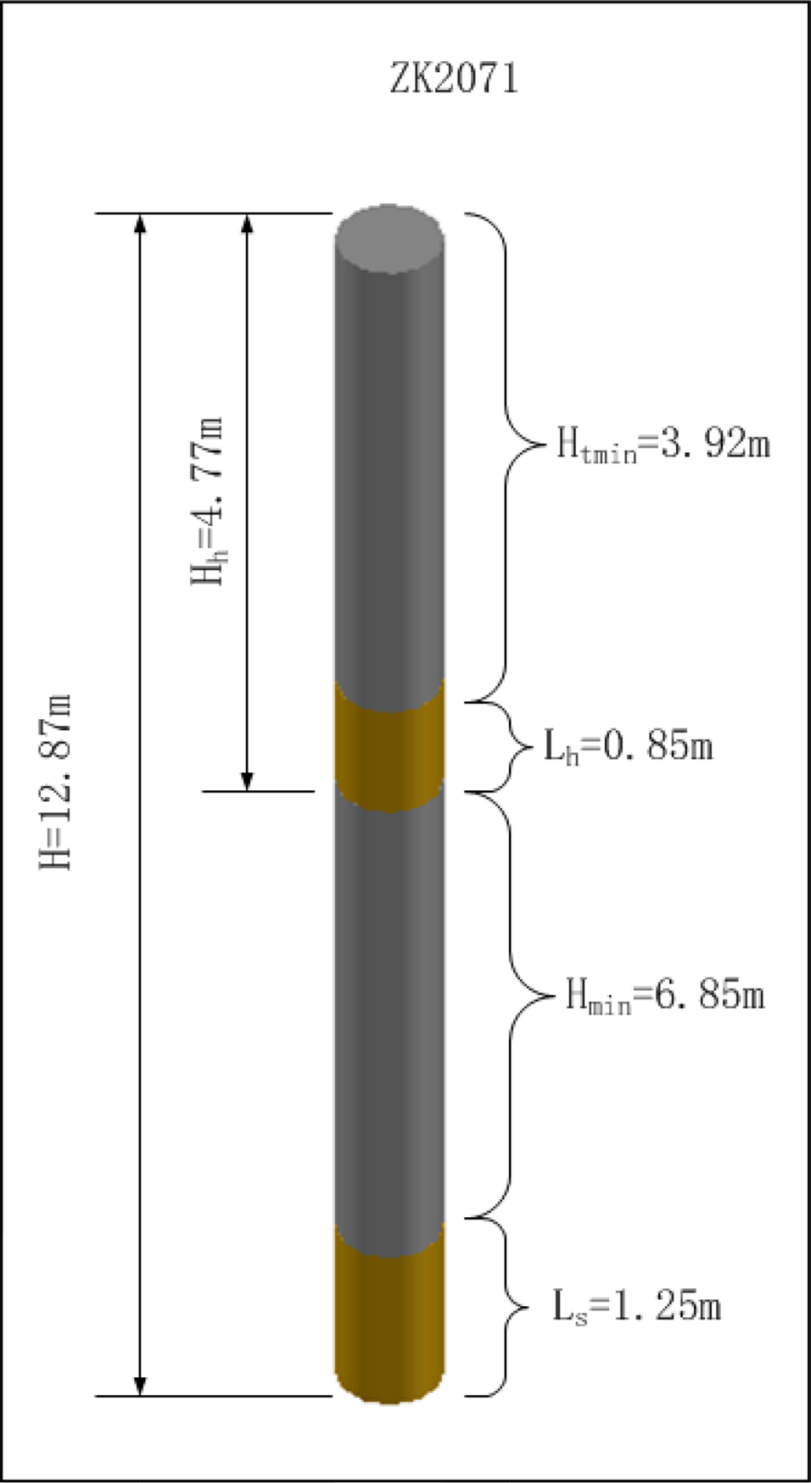
Structure of blasthole ZK2071 segmented charge.

This example calculates the hard rock charge depth *H*_*h*_ and intermediate air filling length *H*_*mid*_ (Stemming 2). According to the hard rock charge depth calculation formula, because of $H_{h} { - }H_{t\min } < L_{h}$, the hard rock charge depth *H*_*h*_ is calculated by Formula (8) to be 4.77 m; then according to the intermediate air filling length calculation Formula (9), the intermediate air filling length *H*_*mid*_ (Stemming 2) is 6.85 m. The calculation results are generated from the blasthole charge table, as shown in Table 4. The three-dimensional diagram of the blasthole ZK2071 segment charge structure is shown in Fig. 13.

**Table 4:** Charge structure of blasthole ZK2071.

Explosive segment	Length (m)	Explosive amount (kg)	Depth (m)	Hole depth (m)	Stemming 1 (m)	Stemming 2 (m)
L_1_	0.68	42.81	4.77	12.87	3.92	6.85
L_2_	1.67	63	12.87

**Figure 14:**
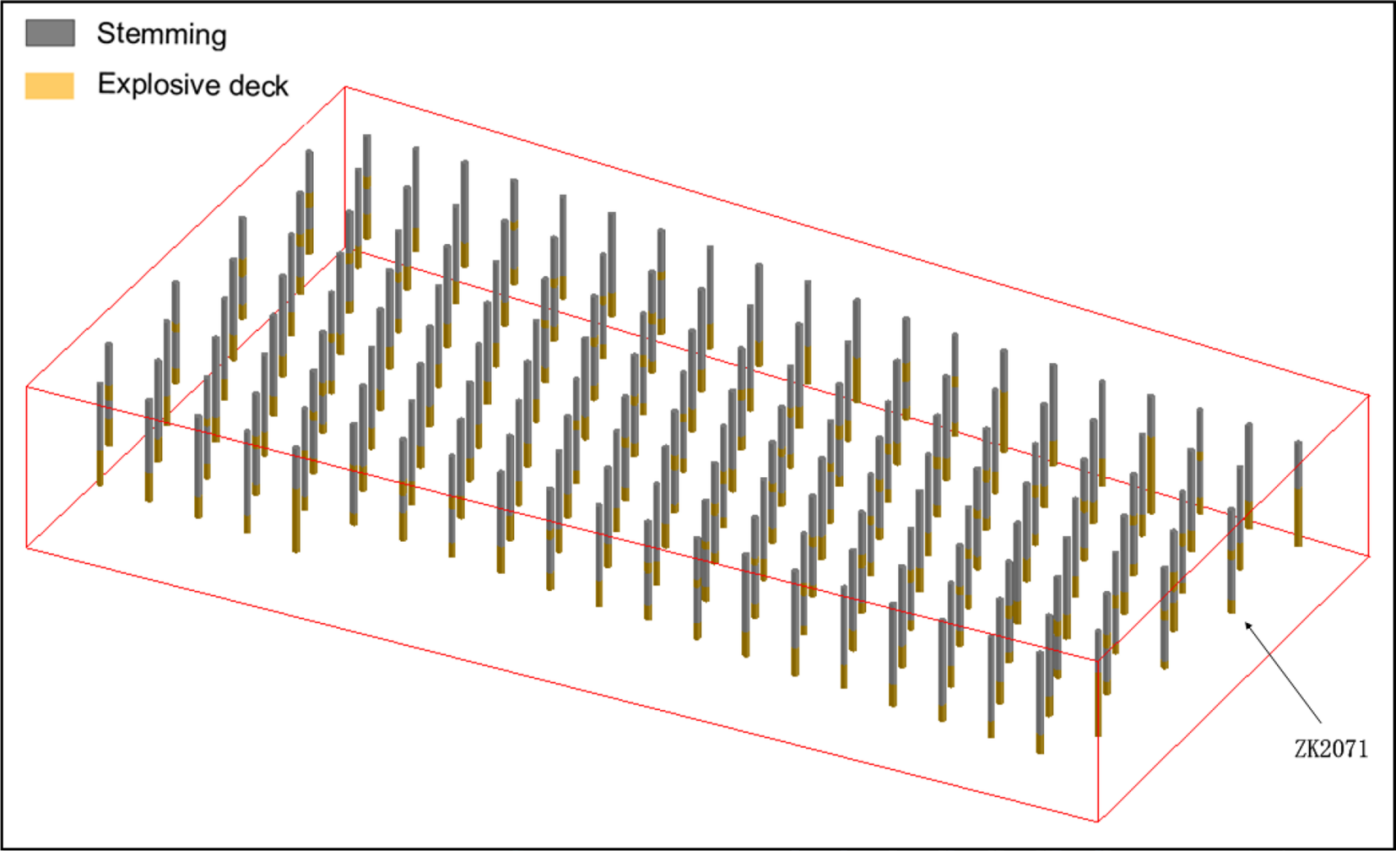
All three-dimensional columnar charge structure.

According to the above calculation steps, the blasthole coordinates represented by the latitude and longitude of the blasthole are converted into X, Y coordinates, all of which and the data of the blasthole are extracted into the blasthole database. We obtained the blasthole charge structure data of all the 165 groups, as shown in Table 5. The three-dimensional columnar charge structure of all blastholes is shown in Fig. 14.

**Table 5:** Blasthole charge structure database.

hold_id	Coordinate			Depth (m)	L_1_ Depth (m)	L_2_ Depth (m)	L_1_ Length (m)	L_2_ Length (m)	Stemming1 (m)
	X	Y	Z						
ZK2023	19,477.49	74,464.34	916.62	12.65	12.65	0.00	5.60	0.00	7.05
ZK2076	19,454.23	74,479.54	917.13	12.87	6.10	12.87	1.10	1.88	5.00
ZK2082	19,438.12	74,511.73	918.73	12.50	6.52	12.50	1.52	2.18	5.00
ZK2153	19,437.24	74,450.91	916.47	12.35	12.35	0.00	2.95	0.00	9.40
ZK2162	19,413.08	74,499.20	917.74	12.83	12.83	0.00	3.69	0.00	9.14
ZK2175	19,424.27	74,461.19	916.97	12.50	6.46	12.50	1.46	1.79	5.00
ZK2179	19,413.53	74,482.66	917.23	12.50	6.76	12.50	1.54	1.32	5.22
…	…	…	…	…	…	…	…	…	…
ZK2187	19,400.56	74,492.94	917.49	12.50	12.50	0.00	4.42	0.00	8.08

According to the design results, the accurate segmented charge design of 165 groups of blastholes in the application example was realized, and the charge and blasting were carried out in the blasting area.

The hole-charge structure design method is realized by Visual C++ 2012 programming. The specific realization method is as follows:

1. ACCESS database operation:
  The ADO method is used to connect the ACCESS database to realize the operation of the database.
2. Calculation and visualization of hole charge structure:
  C++ programming to realize all the calculation work in the design method of the hole charge structure. Based on ObjectARX 2016, the secondary development of AutoCAD 2016 was carried out to realize the three-dimensional columnar display of holes and the visualization of hole charge structure.

### Blasting effect statistics and economic benefit analysis

Based on the lithology distribution, the precise charge experiment of the blast hole was carried out, and the normal blasting design method was also carried out in the field, and the blasting results and economic benefits were analyzed respectively.

1. Analysis of Blasting Results.
  The effect after the normal blasting design:
  - After blasting, there was a large range of irregular uplift in the blasting area, the height of the blasting pile was more than 2 m, the blasting pile was too long and the buried channel was more than half; the explosion was not loose enough, and the machinery was not smooth in the operation process ;
  - During the mechanical cleaning and blasting operation in the blasting area, four large boulders appeared in the middle of the open-pit mine bench, and no part of the rock above 0.5 m appeared on the bench. The bottom of the bench appeared three areas above 4 m^2^, height more than 1.5 m. In order to solve the large boulder in the middle of the bench, the small hole drilling was used for secondary blasting fragmentation;
  - In the blasting process, the working area 3 km away from the blasting operation surface had obvious seismic feeling.
    The effect of hole charge structure design based on lithology distribution:
    - After blasting, the height of the blasting pile in the blasting area did not exceed 2 m, and the buried road before the blasting pile did not exceed one third; and loose burst ;
    - During the mechanical cleaning and blasting operation, there was no large boulder in the middle of the bench, and no rock appeared in the upper part of the bench ; at the bottom of the bench, there was a rock with an area of more than 2 m^2^ and a height of more than 0.8 m, which had little effect on the cleaning operation.
    - In the process of blasting, the seismic sensation in the working area about 3 km away from the blasting working face was obviously lower than that of the normal blasting design.
2. Economic benefit analysis.
  - Normal blasting operation consumption.
    The blasting volume was 129,857.41 m^3^, the number of perforations was 142, and the average hole depth was 12.5 m. The consumption of rock emulsion explosive was 1420 kg, and that of ammonium oil explosive was 15,136.83 kg. The average unit consumption was 0.1275 kg/m^3^. 284 non-conductive detonators were used in one section of the hole, and 142 non-conductive detonators were used in four sections outside the hole. The unit price of statistical consumables are shown in Table 6^33^.

**Table 6:** Blasting materials consumables price.

Name of explosive equipment consumablesme	Rock emulsion explosive/kg	Ammonium oil explosive/kg	1-segment 15 m non-conductive detonators	4-segment 10 m non-electric conduit detonators	Perforation cost/m
Unit price/yuan	12	5	11	12	6
    It is calculated that the cost of waste stripping per cubic meter of the normal blasting operation is 0.8659 yuan.
  - Explosion operation consumption after hole charge structure design based on lithology distribution.
    The blasting volume was 133,965.32 m^3^, the number of perforations was 165, and the average hole depth was 12.55 m. The consumption of rock emulsion explosive was 2505.15 kg, and the consumption of ammonium oil explosive was 13,369.74 kg. The average unit consumption was 0.1185 kg/m^3^. 330 non-conductive detonators with 7 sections in the hole and 165 non-conductive detonators with 3 sections outside the hole were used. The unit prices of statistical consumables are shown in Table 7^33^.

**Table 7:** Blasting materials consumables price.

Name of explosive equipment consumablesme	Rock emulsion explosive/kg	Ammonium oil explosive/kg	3-segment 10 m non-conductive detonators	7-segment 15 m non-electric conduit detonators	Perforation cost/m
Unit price/yuan	12	5	10	13	6
    Through calculation, the cost per cubic meter of waste stripping in blasting operation is 0.8311 yuan after the design of charge structure based on lithology distribution.
    The practical application results show that the cost of waste stripping per cubic meter after blasting is reduced by 0.0339 yuan per cubic meter compared with the normal blasting design. At the same time, the cost of secondary crushing of large blocks and the cost of treating the bottom of the bench are reduced. The blasthole charge structure design method based on lithology distribution can effectively improve the blasting effect , reduce the blasting cost and at the same time reduce the vibration sensation.

## Conclusion

Use intelligent drilling rigs to obtain the lithology distribution in the blasthole, and divide the blasthole rock strata into two groups in terms of the hardness of the lithology: hard rock and soft rock, in this way, the amount of explosive and the length of the charge required for the soft rock group and hard rock group are calculated respectively. According to the thickness of the hard rock and the charge position of the hard rock, the blasthole charge structure is designed, and the following conclusions are obtained:

1. Use intelligent drilling rigs to obtain the lithology distribution data of the blastholes. In terms of the hardness of the rock, the blasthole lithology is divided into 7 categories, and the adjacent rock strata with similar lithologies are combined and grouped into two: soft rocks and hard rocks;
2. A method for the charge structure is proposed depending on the thickness of the hard rock and the location of the hard rock, and the charge amount and charge length of the blasthole is calculated, and the position and size parameters of the blasthole segment charge are determined, and the charge structure table of the hole is established;
3. The use of the C++ programming language realized the blasthole charge structure design method. This method was successfully applied in the Shengli open-pit coal mine in Xilinhot, Inner Mongolia Autonomous Region, China. The application results show that compared with the traditional charge structure design method, the hole charge structure design method can effectively improve the blasting effect, enhance the rock crushing effect, and reduce the ground vibration.
